# Chemoradiotherapy and Lymph Node Metastasis Affect Dendritic Cell Infiltration and Maturation in Regional Lymph Nodes of Laryngeal Cancer

**DOI:** 10.3390/ijms25042093

**Published:** 2024-02-08

**Authors:** Kanako Kawasaki, Keita Kai, Akimichi Minesaki, Sachiko Maeda, Moriyasu Yamauchi, Yuichiro Kuratomi

**Affiliations:** 1 Department of Pathology & Microbiology, Faculty of Medicine, Saga University, Saga 849-8501, Japan; 21624002@edu.cc.saga-u.ac.jp (K.K.); 21624016@edu.cc.saga-u.ac.jp (S.M.); 2 Department of Otolaryngology—Head & Neck Surgery, Faculty of Medicine, Saga University, Saga 849-8501, Japan; sm6511@cc.saga-u.ac.jp (A.M.); yamamori@cc.saga-u.ac.jp (M.Y.); kuratomy@cc.saga-u.ac.jp (Y.K.); 3 Department of Pathology, Saga University Hospital, Saga 849-8501, Japan

**Keywords:** dendritic cell, CD1a, S100, lymph node, chemoradiotherapy

## Abstract

Dendritic cells (DCs) are the most specialized antigen-presenting cells, and lymph nodes (LNs) play an important role in the DC-mediated T-cell response. We evaluated the infiltration of CD1a-positive DCs (CD1a-DCs), i.e., immature DCs, and S100-positive dendritic cells (S100-DCs), a mixture of immature and mature DCs, in 73 cases of laryngeal cancer and its regional LNs. Among them, 31 patients underwent radiotherapy (RT) or chemoradiotherapy (CRT) prior to surgery. No significant difference was found for CD1a-DC infiltration in the primary tumors, metastatic LNs and non-metastatic LNs, while S100-DCs were significantly fewer in number in the primary tumors and metastatic LNs compared to non-metastatic LNs. The cases which showed a high infiltration of S100-DCs in the metastatic LNs appeared to show a favorable prognosis, although statistical significance was not reached. In the RT/CRT group, the infiltration of the CD1a-DCs and S100-DCs was less in the primary tumors and metastatic LNs compared to the treatment-naive group. Conversely, the RT/CRT group showed higher CD1a-DC and S100-DC numbers in the non-metastatic LNs compared to the treatment-naïve group. Thus, DC maturation in metastatic LNs plays an important role in tumor immunity in laryngeal cancer, and the infiltration of DCs into the primary tumor and metastatic LNs is impaired by RT/CRT.

## 1. Introduction

Laryngeal cancer is a common malignancy of the head and neck. Although the prognosis of early laryngeal cancer is favorable, that for advanced laryngeal cancer is poor, despite much progress being made with regard to multidisciplinary therapy, such as the combined use of chemoradiotherapy and surgery [1]. For patients who have lost voice function due to laryngectomy, electrolarynx, esophageal speech and tracheoesophageal speech are used to supplement voice function and maintain their quality of life [2].

In Japan, laryngeal cancer is the second most common head and neck squamous cell carcinoma. A total of 5111 new cases were diagnosed in 2019, with a male-to-female ratio of 11:1, predominantly in males; the 5-year survival rate is 81.0% [3]. It is overwhelmingly more common in males, which may be due to smoking [4]. However, smoking rates have been reported to be declining in younger patients worldwide, with less difference between younger males and females than in elderly patients. Factors other than smoking, such as laryngopharyngeal reflux and HPV infection, have been pointed out in young-onset patients [5].

The treatment strategy for laryngeal cancer is different according to the tumor stage. Local resection or radiation therapy is the first choice for patients with early-stage cancer [6], but for advanced cancer, larynx-preserving surgery or total laryngectomy is performed. Cervical LN dissection may be carried out depending on the presence of LN metastases. Chemoradiotherapy is usually required when the postoperative pathology shows positive margins or LN metastasis with extracapsular extension [7]. If surgery is not an option, chemoradiotherapy is the first treatment of choice, but if residual tumor is observed after treatment, salvage surgery is performed [8]. In cases with T4 or LN metastases, induction chemotherapy should be given first, followed by chemoradiotherapy if a response is elicited. If there is no response, surgery is considered [9]. Thus, there is a wide range of treatment options for laryngeal cancer, which may be modified by the patient’s desire to preserve their larynx or surgical risk due to an underlying condition. Currently, there is no unified treatment strategy for laryngeal cancer, and it is often difficult to decide between surgery and chemoradiotherapy. Regarding chemotherapy, the first choice for induction chemotherapy is a three-drug combination of cisplatin + docetaxel + 5-FU (5-fluorouracil). Instead of cisplatin, carboplatin or cetuximab may also be selected [10]. For recurrent, metastatic or unresectable cases, a Combined Positive Score (CPS) is calculated based on PD-L1 (22C3) immunostaining results. If positive, chemotherapy with pembrolizumab alone or pembrolizumab + cisplatin + 5-FU is selected; if negative, chemotherapy with cisplatin + 5-FU + cetuximab is given [11].

In recent years, immune checkpoint inhibitors have contributed to the improved prognosis of laryngeal cancer. The importance of tumor immunity research and immunotherapy is expected to increase. However, there are many unknowns about the immune microenvironment of laryngeal cancer, and elucidating the immune environment may lead to the development of effective immunotherapy and improved treatment outcomes for laryngeal cancer.

DCs play important roles in cancer immune responses. First, they phagocytose necrotic cancer cell antigens. Subsequent T-cell responses require signals, such as inflammatory cytokines released by tumor cells to prevent immune tolerance to the tumor antigen in the periphery. DCs then present the antigen captured on the major histocompatibility complex (MHC) I or MHC II molecules to T-cells. The T-cell response to the cancer-specific antigen is then primed and activated. The ratio of effector T-cells to regulatory T-cells is determined and influences the outcome. Activated effector T-cells migrate to and infiltrate tumor sites, where they interact with antigens bound to T-cell receptors and MHC I molecules, which then specifically recognize and bind to cancer cells, destroying them [12]. T-cells recognize lipid antigens in a complex with CD1 antigen-presenting molecules. Humans have five CD1 genes encoding five proteins: CD1a, b, c, d and e. The CD1 isoforms overlap but have distinct lipid-binding specificities, which affect the repertoire of lipid antigens that stimulate T-cells. CD1a expression declines as DCs mature and acquire the ability to present antigens [13,14].

Several studies on various types of carcinomas have focused on the infiltration of DCs into tumor tissue [15,16,17,18,19,20,21,22], but no unanimous opinion has been reached. In a previous study conducted in our laboratory, CD1a-DCs were found to be associated with unfavorable clinical outcomes in patients with advanced laryngeal cancer who had undergone total laryngectomy as the initial treatment [23]. However, only a small number of studies have focused on DC infiltration into laryngeal cancer tissue, but the results differed [24,25,26,27,28,29]; thus, the role of DC infiltration in laryngeal cancer remains unclear.

LNs play a very important role in the DC-mediated T-cell response. After antigen phagocytosis, DCs are activated and express C-chemokine receptor 7 (CCR7), a specific chemokine receptor that promotes their migration to LNs, and they are directed by chemokines to the draining lymph vessels and to the T-cell areas of LNs, where they initiate T-cell responses [30,31]. To the best of our knowledge, there has been no specific study that has focused on DC infiltration into the LNs of cancer patients.

In the present study, the aim was to elucidate the trend of DCs in tumor immunity in laryngeal cancer by analyzing DC infiltration into the regional LNs and tumor tissue. Furthermore, we also evaluated the status of DC infiltration after radiotherapy and/or chemoradiotherapy.

## 2. Results

### 2.1. Clinicopathological Features of 73 Patients with Laryngeal Cancer

The clinical and pathological findings of the cohort of 73 laryngeal cancer patients composed of 70 males (95.9%) and 3 females (4.1%) are summarized in Table 1. The median age at initial diagnosis was 68.9 years. The primary tumor sites were glottic in 36 (49.3%) patients, supraglottic in 36 (49.3%) and subglottal in 1 (1.4%). The T-stages at initial diagnosis were T1 in 8 (11.0%), T2 in 21 (28.8%), T3 in 21 (28.8%) and T4 in 23 (31.5%) patients, respectively. Forty-six (37.0%) patients had metastatic LNs, and one patient had no non-metastatic LN specimens. The stages at initial diagnosis were Stage I, 5 patients (6.8%); Stage II, 11 patients (15.1%); Stage III, 16 patients (21.9%); and Stage IV, 41 patients (56.2%). The histology of the patient tumors was all squamous cell carcinoma (SCC), except for one case of carcinosarcoma containing an SCC component.

Forty-two (57.5%) patients underwent surgical resection as their initial treatment. Two (2.7%) patients underwent preoperative radiotherapy (RT) and then underwent surgery, and 29 (39.7%) were given chemoradiotherapy (CRT) before surgery. The primary tumor was not detectable after CRT in eight cases. In these cases, the biopsy specimens obtained prior to CRT were defined as treatment-naïve primary tumor tissue. Thus, 50 cases of primary tumor tissue were finally evaluated as treatment naïve.

### 2.2. Assessment of DCs in Primary Tumors and Regional LNs

**Figure 1:**
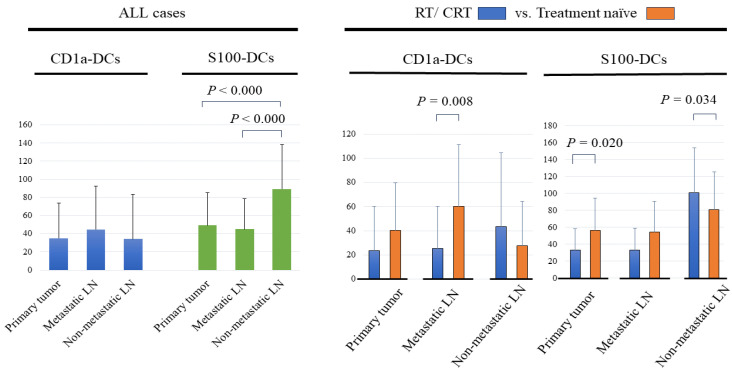
The average number of infiltrating DCs in primary tumors, metastatic LNs and non-metastatic LNs, respectively. The left figure shows the evaluation for all patients (*n* = 73), the right figure shows the evaluation for patients after RT/CRT (*n* = 31) vs. treatment-naïve patients (*n* = 42).

The results of the CD1a-DCs and S100-DCs, which were evaluated for each primary tumor, metastatic LNs and non-metastatic LNs, are shown in Figure 1. The average number ± standard deviation (SD) of the CD1a-DCs, which are considered to be immature DCs, in primary tumors was 35.1 ± 38.9 (median: 21). The average number of CD1a-DCs in the metastatic LNs and non-metastatic-LNs was 44.9 ± 47.7 and 34.3 ± 49.0, respectively. There were no significant statistical differences in the numbers of CD1a-DCs found in the primary tumors, metastatic LNs and non-metastatic LNs.

The average numbers ± SD of the S100-DCs, which are considered to be a mixture of immature DCs and mature DCs, in primary tumors was 49.2 ± 36.0 (median: 43). The average numbers of the S100-DCs in the metastatic LNs and non-metastatic LNs were 45.1 ± 33.7 and 89.5 ± 49.1, respectively. The numbers of the S100-DCs in the non-metastatic LNs were significantly greater than in the metastatic LNs (*p* < 0.000) or primary tumors (*p* < 0.000).

### 2.3. Comparison of DCs in Primary Tumors and Regional LNs between the RT/CRT and Treatment-Naïve Groups

In the analysis of the RT/CRT and treatment-naïve groups, the average number ± SD of CD1a-DCs in the primary tumors, metastatic LNs and non-metastatic LNs of the RT/CRT group were 23.4 ± 36.7, 25.1 ± 35.2 and 43.2 ± 61.2, and in the treatment-naïve group were 40.4 ± 39.0, 60.1 ± 51.0 and 27.6 ± 36.7, respectively. The numbers of the CD1a-DCs in the primary tumors and metastatic LNs in the RT/CRT group were fewer than in the treatment-naïve group. A statistically significant difference was found for the metastatic LNs (*p* = 0.008) but not for the primary tumors (*p* = 0.128). Conversely, the number of CD1a-DCs in the non-metastatic LNs in the RT/CRT group appeared to be greater than in the treatment-naïve group, but statistical significance was not reached (*p* = 0.140).

The average number ± SD of S100-DCs in the primary tumors, metastatic LNs and non-metastatic LNs of the RT/CRT group were 33.0 ± 25.5, 33.2 ± 26.0 and 101.0 ± 52.8, and for the treatment-naïve group were 56.6 ± 37.9, 54.3 ± 36.4 and 80.8 ± 44.7, respectively. The numbers of the S100-DCs detected in the primary tumors and metastatic LNs in the RT/CRT group were fewer than in the treatment-naïve group. Statistical significance was found after an analysis of the primary tumor (*p* = 0.020) but not for the metastatic LNs (*p* = 0.077). Conversely, the numbers of the S100-DCs in the non-metastatic LNs in the RT/CRT group was significantly greater than in the treatment-naïve group (*p* = 0.034).

### 2.4. Clinicopathological Features per CD1a-DCs Infiltration in Primary Tumors, Metastatic LNs and Non-Metastatic LNs

In the primary tumors, the patient cohort was divided into a CD1a-low group (*n* = 37) and a CD1a-high group (*n* = 36) by a cut-off value determined by the median. No significant differences were found in age, gender and the TNM stage between the CD1a-low and CD1a-high groups. The patients who received RT/CRT had significantly fewer CD1a-DCs (*p* = 0.043).

For the analysis of the metastatic LNs, the patient cohort was divided into a CD1a-low group (*n* = 23) and a CD1a-high group (*n* = 23) by the same cut-off value for the primary tumors. No significant differences were found with regard to age, gender and the TNM stage. The patients who received RT/CRT had significantly fewer CD1a-DCs (*p* = 0.036).

For the analysis of the non-metastatic LNs, the patient cohort was divided into a CD1a-low group (*n* = 40) and a CD1a-high group (*n* = 32) according to the cut-off. The non-metastatic LNs of the older patients tended to have fewer CD1a-DCs (*p* = 0.074). However, no significant differences were observed in age, gender, the TNM stage or RT/CRT (Table 2).

### 2.5. Clinicopathological Features per S-100 DC Infiltration in Primary Tumors, Metastatic LNs and Non-Metastatic LNs

According to the cut-off value determined by the median value for the primary tumor, the patient cohort was divided into an S100-low group (*n* = 37) and an S100-high group (*n* = 36). No significant differences were found with regard to age and the TNM stage in the S100-low and S100-high groups. The patients who received RT/CRT had significantly fewer S100-DCs (*p* = 0.011).

In the analysis of the metastatic LNs, the patient cohort was divided into an S100-low group (*n* = 25) and an S100-high group (*n* = 21) according to the same cut-off value for the primary tumors. No significant difference was found with regard to age, gender or the TNM stage. The patients who received RT/CRT had significantly fewer S100-DCs (*p* = 0.019).

For the analysis of the non-metastatic LNs, the patient cohort was divided into an S100-low group (*n* = 14) and an S100-high group (*n* = 58) according to the cut-off. In the non-metastatic LNs, the patients who had distant metastasis had significantly fewer S100-DCs (*p* = 0.036). No significant difference was found for age, gender, the TNM stage or RT/CRT (Table 3).

### 2.6. Kaplan–Meier Survival Curves According to the Infiltration of CD1a- and S100-DCs

**Figure 2:**
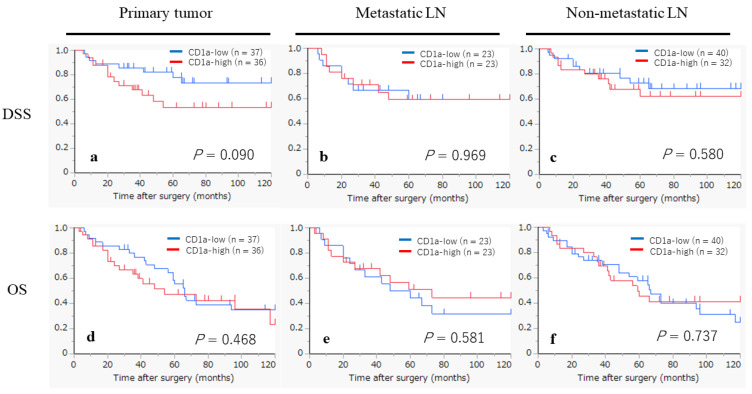
Kaplan–Meier survival curves according to CD1a-DCs infiltration status in primary tumor, metastatic LNs and non-metastatic LNs. (**a**–**c**) Kaplan–Meier survival curves by disease-specific survival (DSS). (**d**–**f**) Kaplan–Meier survival curves by overall survival (OS).

The Kaplan–Meier curves, based on the status of CD1a-DCs infiltration, are shown in Figure 2. In the primary tumors, the CD1a-high group appeared to exhibit a worse prognosis in terms of the disease-specific survival (DSS), although statistical significance was not achieved (*p* = 0.090). However, no tendency was observed in the analyses of the overall survival (OS) for the primary tumors (*p* = 0.468), DSS in the metastatic LNs (*p*= 0.969) and non-metastatic LNs (*p* = 0.580), or for each analysis of the OS for the primary tumors (*p* = 0.468), metastatic LNs (*p* = 0.581) and non-metastatic LNs (*p* = 0.737).

**Figure 3:**
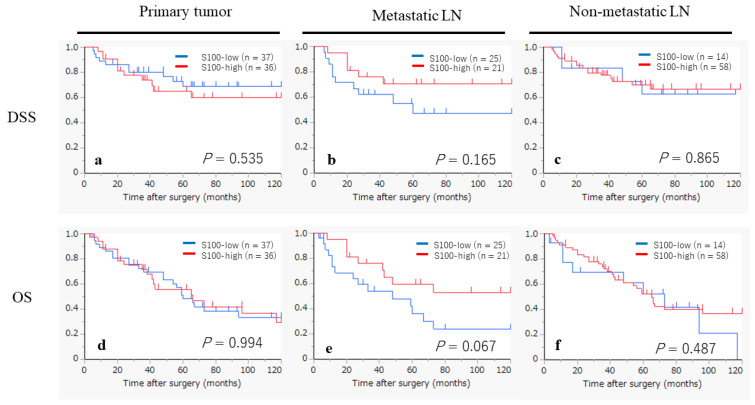
Kaplan–Meier survival curves according to the S100-DC infiltration status in primary tumors, metastatic LNs and non-metastatic LNs. (**a**–**c**) Kaplan–Meier survival curves by disease-specific survival (DSS). (**d**–**f**) Kaplan–Meier survival curves by overall survival (OS).

The Kaplan–Meier curves, based on the status of the S100-DC infiltration, are displayed in Figure 3. In the metastatic LNs, the S100-high group appeared to show a favorable prognosis in terms of the DSS and OS, although statistical significance was not reached (*p* = 0.165, *p* = 0.067, respectively). However, no tendency was observed in the analysis of the DSS for the primary tumors (*p* = 0.535) and non-metastatic LNs (*p* = 0.865), or in the analysis of the OS for the primary tumors (*p* = 0.994) and non-metastatic LNs (*p* = 0.487).

### 2.7. Univariate Analyses for DSS and OS in All Patients (n = 73)

The results of the univariate analyses for the DSS and OS in all the patients are summarized in Table 4. The only factor that was significantly correlated with the DSS was the N stage (*p* = 0.025). The factors that were significantly correlated with the OS were the T stage (*p* = 0.031) and the N stage (*p* = 0.037). The status of both the CD1a-DC and S100-DC infiltration in each primary tumor, metastatic LN and non-metastatic LN showed no significant correlation with neither the DSS nor OS, although tendencies were observed in the DSS for CD1a-DC infiltration in the primary tumors (*p* = 0.098), the DSS for S100-DC infiltration in the metastatic LNs (*p* = 0.177, and the OS for S100-DC infiltration in the metastatic LNs (*p* = 0.077).

## 3. Discussion

DCs are derived from common myeloid progenitors (CMPs) in the bone marrow and comprise two subtypes. In inflammatory conditions, they differentiate into monocytes and then into monocyte DCs through the expression of the transcription factor Nur77. In the absence of Nur77, CMPs differentiate into dendritic cell progenitors, which differentiate into plasmacytoid DCs (pDCs) or conventional DCs (cDCs). cDCs are immature at first but can differentiate into mature DCs following injury or exposure to pathogen-associated factors or inflammatory cytokines. DCs express CCR7 and migrate to the LNs. In the LNs, mature DCs activate naive T-cells to initiate an immune response [32,33].

Several factors have been implicated in DC differentiation and maturation [34]. Several cytokines affect DCs: IL-6 inhibits DC differentiation and maturation [35,36] and IL-10 inhibits DC differentiation, maturation and certain functions [37,38]. M-CSF inhibits their differentiation into DCs from CD34-positive CMPs [35]. GM-CSF produced by tumors has an inhibitory effect on immature DCs [39], while VEGF inhibits the differentiation of DCs and affects the differentiation of the multiple hematopoietic lineage [40]. These research findings raise the possibility that the activation and maturation of DCs are affected by various cytokines and that not all tumor-infiltrating DCs function as antigen-presenting cells. It is possible that the maturation, activation and T-cell response of DCs may be significantly affected by the histological type or by the progression of the tumor.

In the present study, the infiltration of CD1a-DCs was not significantly different in the primary lesion, metastatic LNs or non-metastatic LNs. However, the infiltration of the S100-DCs was significantly different: the infiltration of the S-100DCs in the non-metastatic LNs was significantly greater than for the primary lesions and metastatic LNs. As S100-DCs are considered to label both immature and mature DCs, these results support the hypothesis that the maturation of DCs was prevented by the presence of cancer cells.

Our previous research has indicated that the infiltration of CD1a-DCs into the primary lesion is associated with an unfavorable prognosis for patients with advanced laryngeal cancer who had undergone a total laryngectomy as their initial treatment [23]. A similar tendency was observed in the present study for a different cohort of patients. The high-CD1a-DC infiltrating group in the primary lesions indicated an unfavorable prognosis compared to the low-CD1a-DC infiltrating group, although statistical significance was not reached. Conversely, while the infiltration of the S100-DCs into the primary lesions and non-metastatic LNs did not affect the prognosis, a higher infiltration of the S100-DCs in the metastatic LNs was correlated with better outcomes. These findings highlight the importance of the maturation of DCs in metastatic LNs for an immune response against laryngeal cancer.

Our study had the following limitations, namely a small sample size, the retrospective nature of the study, the lack of follow-up data and that the assessment of the mature DCs was only an indirect marker of S100. However, the results produced important hints for understanding the immune response mediated by DCs in laryngeal cancer. It is likely that the cancer cells play some important roles in both the infiltration and maturation of DC at the primary sites and in metastatic LNs. Unraveling the mechanism of the induction and maturation of DCs by cancer cells may well lead to the development of new immunotherapies. Therefore, the analysis of various cytokines and chemokines in cancer tissue that are associated with the infiltration and maturation of DCs is an important research area, as well as the examination of the characteristics of CD1a-DC regarding their interaction with cancer cells. This research will provide new insights into our understanding of cancer immune responses mediated by DCs.

It has been shown that antigen phagocytosis and the maturation of DCs are processes affected by RT and chemotherapy [41,42,43,44]. Radiotherapy, in particular, has been reported to have an inhibitory effect on DC functions, suggesting that the presence or absence of radiotherapy may make a difference to DC infiltration and maturation [45,46].

RT exerts its therapeutic effect through DNA damage. It is now recognized that the nucleic acid species produced from this DNA damage are inflammatory and potentially immunogenic. RT enhances antigenicity through the release of antigens associated with tumor cell death, radiation-induced neoantigens and the upregulation of MHC-I molecules. On the other hand, RT initiates immunoregulatory and homeostatic actions that reduce the functions of DCs in the tumor microenvironment. In short, RT promotes antitumor immunity through DCs while simultaneously counteracting their functions [47,48,49,50].

The present research also focused on the status of DC infiltration and maturation in primary tumors and LNs after RT/CRT. Our results indicated that the infiltration of CD1a-DCs were fewer in number in the primary lesions and metastatic LNs in the RT/CRT group compared to the treatment-naïve group. Conversely, the RT/CRT group showed a higher level of CD1a-DC infiltration into the non-metastatic LNs than the treatment-naïve group. Similar results were also obtained in the analyses of the S100-DCs. These results support the following working hypothesis: the ability of DC induction in cancer cells and the surrounding stroma is impaired by necrosis and/or the degeneration of the tumor cells and microenvironmental changes due to RT/CRT. On the other hand, the ability for DC induction remains in non-metastatic LNs, which are relatively unaffected by RT/CRT. The infiltration of DC numbers into non-metastatic LNs, both CD1a-DCs and S100-DCs, was higher in the RT/CT cases than in the treatment-naïve cases. This result was also predictable considering the enhanced immune reaction elicited by RT/CRT.

In conclusion, our study suggested that DC maturation in metastatic LNs plays an important role in tumor immunity in laryngeal cancer. Additionally, the results confirmed DC induction in tumor tissue is impaired by RT/CRT in clinically resected specimens. Understanding these phenomena could open avenues for novel immunotherapies. The further accumulation of clinicopathological and basic research data and validation studies using a larger cohort will be necessary to clarify the mechanisms underlying tumor immune responses involving DCs.

## 4. Materials and Methods

### 4.1. Patients

**Figure 4:**
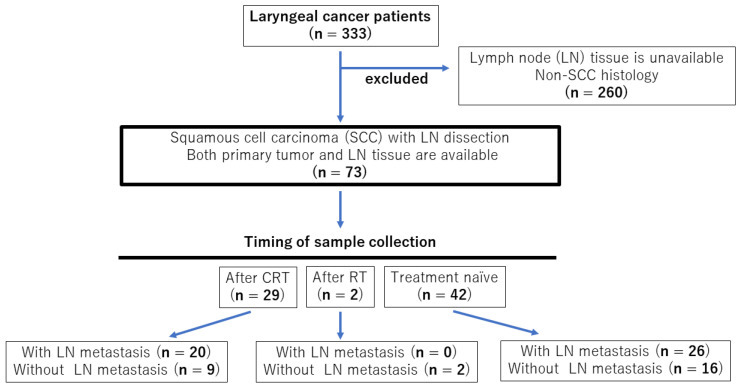
Flow chart showing case selection criteria.

A total of 333 patients with laryngeal cancer treated at Saga University Hospital between 2000 and 2020 were initially enrolled in this study. Among these, cases without lymphadenectomy or lymph node biopsy, and those with histological types other than squamous cell carcinoma were excluded. Finally, 73 patients were enrolled (Figure 4). Comprehensive informed consent for the use of resected tissue for this research was obtained from all patients, and the study protocol was approved by the Ethics Committee of Saga University (2023-02-R-09).

### 4.2. Immunohistochemistry

Immunohistochemistry (IHC) of CD1a and S-100 was carried out on representative primary tumors and regional LNs. In the cases with nodal metastasis, both representative metastatic and non-metastatic LNs were subjected to IHC. For patients without nodal metastasis, only non-metastatic LNs were analyzed using IHC. As one patient had no non-metastatic LNs, only metastatic nodes were subjected to IHC in that case. The specimens were sectioned into 4 µm slices from Formalin-Fixed Paraffin-Embedded (FFPE) blocks. The primary antibodies used were CD1a (Clone 010, prediluted; Dako, Glostrup, Denmark), and S100 (GA50461–2 J; prediluted; Dako). IHC was performed using an Autostainer plus automatic stainer (Dako). The Envision System (Dako) was employed as the secondary antibody. Specimens on slides were visualized by diaminobenzidine tetrahydrochloride and nuclei were counterstained with hematoxylin.

### 4.3. Assessment of CD1a- and S100-DCs

**Figure 5:**
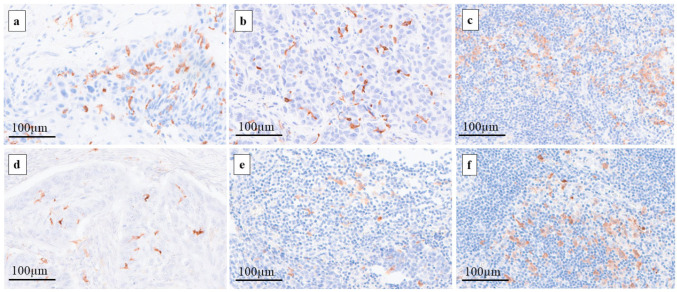
Immunohistochemistry of CD1a (**a**–**c**) and S100 (**d**–**f**). Original magnification of each photo was ×200. (**a**,**d**) Primary tumor, (**b**,**e**) metastatic LN, (**c**,**f**) non-metastatic LN.

The IHC sections were scanned and converted to digital slides using a NanoZoomer S360 (Hamamatsu Photonics, Shizuoka, Japan). CD1a-DCs and S100-positive DCs (S100-DCs) were evaluated in 3 random hot spots at a magnification of ×100 (Figure 5). The median values of CD1a-DCs and S100-DCs in the primary tumors were used as the cut-off values and the patient cohort was divided into a high group and a low group. The same cut-off value determined for the primary tumors was used in the evaluation of LNs. Patients were also divided into high and low groups according to the degree of DC infiltration into LNs.

### 4.4. Statistical Analysis

All statistical analyses were performed using JMP Pro 13.1.0 software (SAS Institute, Cary, NC, USA). For comparisons between two groups, Student’s *t*-test (two-tailed) was used for comparison of the age. Fisher’s exact test (two-tailed) was used for comparison of other factors. Univariate analyses were performed using the Cox proportional hazard model.

Disease-specific survival (DSS) was defined as the period from surgery to cancer-related death or the last follow-up. Overall survival (OS) was defined as the period from surgery to death or the last follow-up. The maximum follow-up period during the study was 120 months, with a median follow-up time of 45.0 months. The survival curve was calculated by the Kaplan–Meier method, and a log-rank test was conducted.

## Figures and Tables

**Table 1:** Clinicopathological features of 73 patients with laryngeal cancer.

Age, Years (Mean ± SD)	68.9 ± 9.4
Sex	
Male/Female	70 (95.9%)/3 (4.1%)
Smoking habit	
Never/Ex/Current	8 (11.0%)/15 (20.5%)/50 (68.5%)
Alcohol abuse	
(−)/(+)	22 (30.1%)/51 (69.9%)
Subsite	
Glottis/Supraglottis/Subglottis	36 (49.3%)/36 (49.3%)/1 (1.4%)
Histology and differentiation	
Well/Mode/Poor/Carcinosarcoma	33 (45.2%)/35 (47.9%)/4 (5.5%)/1 (1.4%)
Primary T stage	
T1/T2/T3/T4	8 (11.0%)/21 (28.8%)/21 (28.8%)/23 (31.5%)
N	
(−)/(+)	27 (63.0%)/46 (37.0%)
Primary M stage	
M0/M1	71 (97.3%)/2 (2.7%)
Stage	
I/II/III/IV	5 (6.8%)/11 (15.1%)/16 (21.9%)/41 (56.2%)
Treatment background	
Surgery without RT/CRT	42 (57.5%)
Surgery after RT	2 (2.7%)
Surgery after CRT	29 (39.7%)

Abbreviations: SD, standard deviation; RT, radiotherapy; CRT, chemoradiotherapy.

**Table 2:** Clinicopathological features per CD1a-DC infiltration.

	Primary Tumor (*n* = 73)			Metastatic LN (*n* = 46)			Non-Metastatic LN (*n* = 72 *)		
	CD1a-Low (*n* = 37)	CD1a-High (*n* = 36)	*p*	CD1a-Low (*n* = 23)	CD1a-High (*n* = 23)	*p*	CD1a-Low (*n* = 40)	CD1a-High (*n* = 32)	*p*
Age, years (mean ± SD)	67.1 ± 8.9	70.5 ± 9.7	0.124	68.3 ± 9.5	67.5 ± 9.8	0.772	70.6 ± 8.4	66.6 ± 10.3	0.074
Sex									
Male	36 (97.3%)	34 (94.4%)	0.615	23 (100.0%)	20 (87.0%)	0.233	39 (97.5%)	30 (93.8%)	0.582
Female	1 (2.7%)	2 (5.6%)		0 (0.0%)	3 (13.0%)		1 (2.5%)	2 (6.3%)	
Primary T stage									
T1/2	17 (46.0%)	12 (33.3%)	0.341	12 (52.2%)	10 (43.5%)	0.768	19 (47.5%)	10 (31.3%)	0.227
T3/4	20 (54.1%)	24 (66.7%)		11 (47.8%)	13 (56.5%)		21 (52.5%)	22 (68.8%)	
N									
N (−)	12 (32.4%)	15 (41.7%)	0.473	0 (0.0%)	0 (0.0%)	n/a	14 (35.0%)	13 (40.6%)	0.634
N (+)	25 (67.6%)	21 (58.3%)		23 (100%)	23 (100%)		26 (65.0%)	19 (59.4%)	
Primary M stage									
M0	36 (97.3%)	35 (97.2%)	1.000	22 (95.7%)	22 (95.7%)	1.000	40 (100.0%)	30 (93.8%)	0.194
M1	1 (2.7%)	1 (2.8%)		1 (4.4%)	1 (4.4%)		0 (0.0%)	2 (6.3%)	
Timing of the resected samples									
After RT or CRT	16 (43.2%)	7 (19.4%)	0.043	14 (60.9%)	6 (26.1%)	0.036	18 (45.0%)	12 (37.5%)	0.632
Treatment naïve **	21 (56.8%)	29 (80.6%)		9 (39.1%)	17 (73.9%)		22 (55.0%)	20 (62.5%)	

* One patient had no non-metastatic LNs. ** Eight cases are biopsy specimens obtained prior to CRT. Abbreviations: DC, dendritic cell; SD, standard deviation; n/a, not available; RT, radiotherapy; CRT, chemoradiotherapy.

**Table 3:** Clinicopathological features per S100-DC infiltration.

	Primary Tumor (*n* = 73)			Metastatic LN (*n* = 46)			Non-Metastatic LN (*n* = 72 *)		
	S100-Low (*n* = 37)	S100-High (*n* = 36)	*p*	S100-Low (*n* = 25)	S100-High (*n* = 21)	*p*	S100-Low (*n* = 14)	S100-High (*n* = 58)	*p*
Age, years (mean ± SD)	68.0 ± 9.3	69.7 ± 9.6	0.445	68.5 ± 10.5	67.2 ± 8.3	0.654	72.1 ± 9.3	68.0 ± 9.4	0.150
Sex									
Male	36 (97.3%)	34 (94.4%)	0.615	24 (96.0%)	19 (90.5%)	0.585	13 (92.9%)	56 (96.6%)	0.483
Female	1 (2.7%)	2 (5.6%)		1 (4.0%)	2 (9.5%)		1 (7.1%)	2 (3.5%)	
Primary T stage									
T1/2	15 (40.5%)	14 (38.9%)	1.000	7 (28.0%)	15 (71.4%)	0.007	5 (35.7%)	24 (41.4%)	0.770
T3/4	22 (59.5%)	22 (61.1%)		18 (72.0%)	6 (28.6%)		9 (64.3%)	34 (58.6%)	
N									
N (−)	15 (40.5%)	12 (33.3%)	0.630	0 (0.0%)	0 (0.0%)	n/a	5 (35.7%)	22 (37.9%)	1.000
N (+)	22 (59.5%)	24 (66.7%)		25 (100%)	21 (100%)		9 (64.3%)	36 (62.1%)	
Primary M stage									
M0	36 (97.3%)	35 (97.2%)	1.000	23 (92.0%)	21 (100.0%)	0.493	12 (85.7%)	58 (100.0%)	0.036
M1	1 (2.7%)	1 (2.8%)		2 (8.0%)	0 (0.0%)		2 (14.3%)	0 (0.0%)	
Timing of resected samples									
After CRT or RT	17 (45.9%)	6 (16.7%)	0.011	15 (60.0%)	5 (23.8%)	0.019	3 (21.4%)	27 (46.6%)	0.131
Treatment naïve **	20 (54.1%)	30 (83.3%)		10 (40.0%)	16 (76.2%)		11 (78.6%)	31 (53.5%)	

* One patient had no non-metastatic LNs. ** Eight cases are biopsy specimens obtained prior to CRT. Abbreviations: DC, dendritic cell; SD, standard deviation; n/a, not available; RT, radiotherapy; CRT, chemoradiotherapy.

**Table 4:** Univariate analyses for DSS and OS in all patients (*n* = 73).

Characteristic	*n*	DSS		OS	
		HR (95% CI)	*p*	HR (95% CI)	*p*
Age			0.683		0.783
≤68 years	38	1		1	
>68 years	35	0.84 (0.35–1.98)		1.09 (0.59–2.03)	
Sex			0.473		0.470
Female	3	1		1	
Male	70	0.48 (0.06–3.58)		0.59 (0.14–2.47)	
T stage			0.109		0.031
T1/T2	29	1		1	
T3/T4	44	2.18 (0.84–5.63)		2.09 (1.07–4.08)	
N stage			0.025		0.037
N0	27	1		1	
N1–3	46	2.96 (1.14–7.67)		1.97 (1.04–3.72)	
M stage			0.050		0.179
M0	71	1		1	
M1	2	4.32 (1.00–18.69)		2.68 (0.64–11.25)	
CD1a-DCs in primary tumor			0.098		0.472
low	37	1		1	
high	36	2.11 (0.87–5.12)		1.25 (0.67–2.35)	
CD1a-DCs in metastatic LN			0.969		0.586
low	23	1		1	
high	23	0.98 (0.36–2.61)		0.80 (0.36–1.79)	
CD1a-DCs in non-metastatic LN			0.582		0.739
low	40	1		1	
high	32	1.28 (0.53–3.08)		0.90 (0.47–1.70)	
S100-DCs in primary tumor			0.538		0.994
low	37	1		1	
high	36	1.31 (0.55–3.09)		1.00 (0.54–1.87)	
S100-DCs in metastatic LN			0.177		0.077
low	25	1		1	
high	21	0.50 (0.18–1.37)		0.47 (0.21–1.08)	
S100-DCs in non-metastatic LN			0.866		0.490
low	14	1		1	
high	58	0.91 (0.30–2.73)		0.77 (0.36–1.62)	

Abbreviations: DSS, disease-specific survival; OS, overall survival; HR, hazard ratio; CI: confidence interval.

## Data Availability

Data contained within the article will be provided by the authors upon reasonable request.

## Abbreviations

CCR7, C-chemokine receptor 7; CMP, common myeloid progenitor; CPS, combined positive score; CRT, chemoradiotherapy; DC, dendritic cell; DSS, disease-specific survival; FFPE, formalin-fixed paraffin-embedded; GM-CSF, granulocyte macrophage colony stimulating factor; IHC, immunohistochemistry; LN, lymph node; M-CSF, macrophage colony stimulating factor; MHC, major histocompatibility complex; Nur77, nuclear receptor 77; OS, overall survival; RT, radiotherapy; VEGF, vascular endothelial growth factor; 5-FU, 5-fluorouracil.
